# Postpartum Remote Health Coaching Intervention for Individuals With a Hypertensive Disorder of Pregnancy: Proof-of-Concept Study

**DOI:** 10.2196/65611

**Published:** 2025-01-08

**Authors:** Jaclyn D Borrowman, Lucas J Carr, Gary L Pierce, William T Story, Bethany Barone Gibbs, Kara M Whitaker

**Affiliations:** 1 Department of Preventative Medicine, Feinberg School of Medicine, Northwestern University, Chicago, IL, United States; 2 Department of Health and Human Physiology, University of Iowa, Iowa City, IA, United States; 3 Department of Community and Behavioral Health, University of Iowa, Iowa City, IA, United States; 4 Department of Epidemiology and Biostatistics, West Virginia University, Morgantown, WV, United States; 5 Department of Epidemiology, University of Iowa, Iowa City, IA, United States

**Keywords:** cardiovascular disease, postpartum, hypertensive disorders of pregnancy, intervention, physical activity intervention, proof-of-concept, cardiovascular health, CVD risk, cardiovascular disease risk, feasibility, acceptability, health coaching, women's health, postnatal

## Abstract

**Background:**

Cardiovascular disease (CVD) is the leading cause of death among women in America. Hypertensive disorders of pregnancy (HDP) negatively impact acute and long-term cardiovascular health, with approximately 16% of all pregnancies affected. With CVD 2‐4 times more likely after HDP compared to normotensive pregnancies, effective interventions to promote cardiovascular health are imperative.

**Objective:**

With postpartum physical activity (PA) interventions after HDP as an underexplored preventative strategy, we aimed in this study to assess (1) the feasibility and acceptability of a remotely delivered PA intervention for individuals with HDP 3‐6 months postpartum and (2) changes in average steps per day, skills related to PA behavior, and postpartum blood pressure (BP).

**Methods:**

A remotely delivered 14-week health coaching intervention was designed based on prior formative work. The health coaching intervention called the Hypertensive Disorders of Pregnancy Postpartum Exercise (HyPE) intervention was tested for feasibility and acceptability with a single-arm proof-of-concept study design. A total of 19 women who were 3‐6 months postpartum HDP; currently inactive; 18 years of age or older; resided in Iowa; and without diabetes, kidney disease, and CVD were enrolled. Feasibility was assessed by the number of sessions attended and acceptability by self-reported satisfaction with the program. Changes in steps achieved per day were measured with an activPAL4 micro, PA behavior skills via validated surveys online, and BP was assessed remotely with a research-grade Omron Series 5 (Omron Corporation) BP monitor.

**Results:**

Participants at enrollment were on average 30.3 years of age, 4.1 months postpartum, self-identified as non-Hispanic White (14/17, 82%), in a committed relationship (16/17, 94%), and had a bachelor’s degree (9/17, 53%). A total of 140 of 152 possible health coaching sessions were attended by those who started the intervention (n=19, 92%). Intervention completers (n=17) indicated they were satisfied with the program (n=17, 100%) and would recommend it to others (n=17, 100%). No significant changes in activPAL measured steps were observed from pre- to posttesting (mean 138.40, SD 129.40 steps/day; *P*=.75). Significant improvements were observed in PA behavior skills including planning (mean 5.35, SD 4.97 vs mean 15.06, SD 3.09; *P*<.001) and monitoring of PA levels (mean 7.29, SD 3.44 vs mean 13.00, SD 2.45; *P*<.001). No significant decreases were observed for systolic (mean –1.28, SD 3.59 mm Hg; Hedges *g*=–0.26; *P*=.16) and diastolic BP (mean –1.80, SD 5.03 mm Hg; Hedges *g*=–0.44; *P*=.12).

**Conclusions:**

While PA behaviors did not change, the intervention was found to be feasible and acceptable among this sample of at-risk women. After additional refinement, the intervention should be retested among a larger, more diverse, and less physically active sample.

## Introduction

Cardiovascular disease (CVD) is the leading cause of death among women in America; with 1 in 4 deaths being preventable [1]. Hypertensive disorders of pregnancy (HDP) are associated with a 2‐4 times greater risk of developing CVD later in life [1,2]. Despite this increased risk for CVD and with 16% of all pregnancies affected, few studies have explored potential interventions for this population after delivery [3]. Consequently, it is imperative that preventative strategies are developed and started as early as possible after delivery to mitigate the risk of future CVD and improve maternal morbidity and mortality.

Cardiovascular changes during pregnancy act as a natural stress test and may uncover an individual’s potential risk of future CVD [4]. Specifically, individuals with HDP are at an immediately increased risk for hypertension (HTN) after delivery [4]. Long-term cardiometabolic health is also impacted by HDP, with an increased risk of future CVD, morbidity, and mortality compared to normotensive pregnancies [5]. The odds of developing HTN at 1 year postpartum are 5 to 7 times greater in hypertensive pregnancies compared to normotensive pregnancies [2,6]. The 5-year likelihood of developing HTN for pregnancies complicated by HDP is 7.1 times greater than for normotensive pregnancies [4]. Even in those not diagnosed with HTN, average blood pressure (BP) is greater in HDP women than in women with normotensive pregnancies.

Low physical activity (PA) is a known modifiable risk factor for CVD, and increasing PA has the potential to improve BP [7]. PA, specifically aerobic exercise, has beneficial effects on mean, systolic BP (SBP), and diastolic BP (DBP) in adults [8,9]. This effect has been observed in pregnant populations as well, with the greatest improvements seen among inactive individuals [10,11]. Despite the known benefits of PA, recent studies suggest moderate to vigorous PA decreases after pregnancy compared to during pregnancy activity levels [12]. The perinatal period, therefore, presents a critical opportunity for behavior change [13-15]. Interventions promoting PA in the postpartum period after HDP are an underexplored primary prevention strategy warranting further investigation.

The main objective of our study was to assess the feasibility and acceptability of a remotely delivered PA intervention for individuals with HDP during the postpartum period. We hypothesized the intervention would be highly feasible and acceptable. Our secondary objectives included assessing changes in average steps per day, skills related to PA behavior, and postpartum BP. We hypothesized that we would observe significant increases in steps, improvements in PA skills, and decreases in postpartum SBP and DBP.

## Methods

### Study Design

A remotely delivered PA intervention for individuals with HDP called the Hypertensive Disorders of Pregnancy Postpartum Exercise (HyPE) intervention, was tested with a single-arm quasi-experimental study design. The Obesity-Related Behavioral Intervention Trials (ORBIT) model was the conceptual framework that was leveraged in the development and subsequent testing of the HyPE intervention [16]. The ORBIT model guides the development of behavioral treatments to prevent or manage chronic disease. This model describes a flexible and iterative process that includes prespecified clinically significant milestones necessary for forward movement through the developmental pathway.

Phase I of the ORBIT model was previously completed by our group, where the design of this intervention was informed by an assessment of the PA determinants, needs, and desired components of a PA intervention of postpartum individuals after HDP [17]. Given phase I was completed successfully, our group subsequently developed the HyPE intervention for proof-of-concept testing (phase IIa of the ORBIT model). Study methods appropriate at this stage include a quasi-experimental, treatment-only design to determine whether the intervention is feasible, acceptable, and can achieve a clinically significant signal on the behavioral risk factor of interest (ie, PA).

### Ethical Considerations

All methods and procedures were approved by an institutional review board prior to the start of the study (IRB #202302470; ClinicalTrials: NCT06019715). Potential participants had a call with a research staff member to review study details, ask questions, and provide informed consent if still interested in enrolling. All data were deidentified and secured. For compensation, participants were provided a Fitbit Inspire 2 (Google) and Omron Series 5 BP monitor. Participants received a US $20 Amazon gift card after completion of half of the study visits and a US $25 Amazon gift card after completion of the entire study.

### Recruitment

Recruitment of currently inactive individuals (defined in this study as achieving <9000 steps on average per day) occurred via mass emails to faculty and staff members of a large midwestern university, targeted emails to participants in the Pregnancy 24/7 Cohort Study, and postpartum medical record searches of individuals who received care at local hospitals and clinics. If participants were interested in the study, the target email contained information, as well as a link to a REDcap (Vanderbilt University) survey for initial screening. If eligible, interested individuals were contacted via phone at their preferred time by a research staff member to describe the study in further detail and answer questions. Subsequently, potential participants were provided the opportunity for participants to provide informed consent if still interested in the study.

Following informed consent, participants started baseline screen procedures involving the assessment of their current PA levels. The threshold of inactivity was set at <9000 steps per day based on a prior study conducted by our group and concerns regarding recruitment within a restricted timeframe [18]. Screening of baseline PA levels was conducted with an activPAL4 micro prior to participation in the first study visit.

### Eligibility

To be included, participants were 3‐6 months postpartum after a pregnancy complicated by HDP; 18 years of age or older; able to speak, read, and write English; lived in Iowa; and owned a smartphone. Exclusions included current enrollment in a PA intervention; achieving >9000 steps at pretesting; inability to walk half block or 2 flights of stairs; physician’s recommendation to limit PA; and current diagnosis and treatment of diabetes, kidney disease, and CVD. All self-report diagnoses of HDP were verified by medical records.

### Intervention

**Figure 1.**
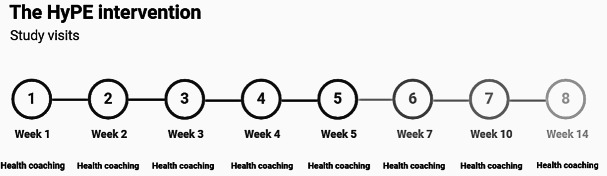
The Hypertensive Disorders of Pregnancy Postpartum Exercise (HyPE) intervention health coaching study timeline. The HyPE intervention was a 14-week health coaching intervention for postpartum individuals after having a pregnancy complicated by a hypertensive disorder. This intervention included 8 total health coaching session aiming to promote physical activity.

The overall goal of the intervention was to increase the average number of steps participants achieved per day, with the specific objective to increase steps by approximately 10% between each session. The intervention consisted of a total of 8 online health coaching sessions, with sessions 1‐5 occurring weekly (ie, the intensive intervention) and subsequent visits 6‐8 occurring with reduced frequency (Figure 1). Participants were provided a Fitbit Inspire 2 to track their progress toward the step goals set with their health coach. Formative work conducted by our group informed the creation of tailored educational postpartum PA intervention, including the use of a health coach, remote delivery approach, selected frequency of study visits, and topics offered to study participants (eg, breastfeeding and PA).

Health coaching was informed by motivational interviewing techniques and conducted by a trained interventionist (principle investigator: JDB). This approach was developed to promote behavioral change and work through ambivalence by supporting an individual’s commitment to change using “change talk” (ie, eliciting the client to express desires, ability, reasons, and need for a change of behavior) [19,20].

### Measures

REDCap was used to build and manage survey assessments [21]. At pre- and posttesting, the following information was collected.

#### Demographics

Questions assessed the individual’s age, race, ethnicity, marital status, education, annual household income, insurance coverage, parity, and employment status. Those currently employed were asked questions assessing job type, hours worked per week, if parental leave was provided, and weeks of parental leave provided.

#### PA Identity

PA identity was assessed with the validated exercise self-identity scale [22]. The 9-item survey assessed various components for how strongly individuals felt exercise was an important component of self. Individuals rated each item on a 7-point Likert scale from “strongly disagree” (0) to “strongly agree” (6) which were summed for a total score (higher scores indicated exercise was a greater component of self).

#### PA Planning and Monitoring

PA planning and self-monitoring behaviors were assessed with the validated 8-item self-monitoring and 6-item action planning questionnaires [23]. Questions were assessed on a 4-point Likert scale from “completely disagree (0)” to “totally agree (3).” Scores were calculated by summing responses; higher scores indicated greater self-monitoring and planning of PA.

#### Program Evaluation

At the conclusion of the intervention, participants responded to a series of closed-ended and open-ended questions assessing their acceptance of the overall program and different components of the intervention including health coaching sessions, perception of PA changes, quality of life, usefulness of tailored intervention materials, the Fitbit, activPAL4 micro use, and home BP monitoring with a 5-point Likert scale from “strongly disagree” (0) to “strongly agree” (4). These questions were adapted from a previously tested intervention [18].

#### PA Behavior

Device-based measurement of PA was conducted with both the activPAL4 micro and the Fitbit Inspire 2. ActivPAL measurement gave research grade assessments of PA at pre- and posttesting while the Fitbit allowed for steps measurement to occur at the end of the intensive intervention (week 5). The activPAL4 micro assessed changes in PA between pre- and posttesting and data from the Fitbit Inspire 2 were used to assess changes in steps during the intervention. The week prior to the first and last health coaching sessions, participants were instructed to wear an activPAL4 micro for 9 consecutive days (2 partial days and 7 full days) that they received via mail along with instructions for the device. They were also provided an activity log to self-report sleep, naps, and nonwear times. The activPAL4 micro was wrapped with retention tape and then adhered to the anterior thigh with transparent dressing (3M Tegaderm Film) for 24 hours a day, to be removed only when swimming to prevent monitor loss.

For assessment of the preliminary efficacy of the intervention, our primary outcome was a change in steps per day measured with the activPAL4 micro. Data collected from the activPAL4 micro were exported as events using PAL technologies software using the VANE algorithm (PAL Technologies). Participants’ self-reported sleep and nonwear times in their activity logs were removed using R (R Core Team) [24]. The processing procedures for this approach are available via the pregnancy247 package located on GitHub [25]. This code generates a figure depicting sitting, standing, or stepping activities, as well as sleep or nonwear periods. Visual inspection of these graphs was conducted to verify the accuracy of reported sleep onset, offset, and naps. Movement behaviors during waking hours on valid days were summarized using the activPAL processing package created by Lynden et al [26]. Total wear time and valid days were defined by at least 5 days of total wear with greater than 10 hours of wear time each day [27].

For the Fitbit Inspire 2, participants were instructed to wear the device every day, only removing it at night to charge. At the end of study participation, all step data collected from the Fitbit were retrieved by the study staff for data analysis. A valid day was defined as achieving >1000 steps [28].

#### Blood Pressure

The protocol for this study’s BP assessment followed the American Heart Association’s monitoring home BP recommendations [26,29]. Participants were provided the validated Omron Series 5 BP monitor at the start of the study [30,31], and received verbal and visual instructions on how to take an accurate reading during visit 1, visit 4, and visit 7. Participants were asked to measure their BP at pretesting, at the end of the intensive intervention (between visits 4 and 5), and a week prior to the final study visit which was at the same time as PA assessments that occurred with both the activPAL4 micro and Fitbit. During each of these assessments, participants were asked to measure their BP twice in the morning and twice at night for 7 consecutive days (ie, 28 total readings). These readings were synced with the Omron Connect app on the participant’s cellular device and shared with the research staff via email. Assessment of appropriate BP technique occurred remotely via Zoom (Zoom Communications) at each study visit with corrections of technique provided when needed.

### Statistical Analysis

All statistical analyses were determined a priori for main objectives and SAS (version 9.4; SAS Institute) was used for analysis. Demographic characteristics were assessed using frequencies and averages. Feasibility was measured by the number of completed sessions out of the total sessions possible. Acceptability was assessed by participants’ self-reported satisfaction and likelihood of recommending the program to others. High feasibility was defined as 75% of all sessions attended. High acceptability was defined by at least 75% of participants responding that they were either satisfied or very satisfied with the program.

To assess changes in PA and BP between timepoints, data from the activPAL4 micro, Fitbit, and Omron series 5 were analyzed using paired *t* tests, Wilcoxon signed-rank, and Fisher exact tests. To estimate the effect size, Hedges *g* statistics were used. Thresholds for small, medium, and large effect sizes were set at 0.2, 0.5, and 0.8, respectively [32]. Linear regression models were used to assess the association of change in PA (average steps per day) and change in BP across the intervention [33]. SBP and DBP were treated as continuous variables and included in separate models. Covariates included in the final model were the participant’s age, annual household income, activPAL wear time, and pretest steps.

Although a proof-of-concept study, a sample size calculation was conducted with a 0.50 effect size, based on preliminary evidence concerning peripartum PA interventions, using 80% power and at an α level of .05. From this, it was estimated that 15 individuals would be needed to detect significant changes in PA. Allowing for 25% drop out, the goal was to recruit 20 participants.

## Results

### Recruitment

Of the 94 individuals who completed the eligibility survey, 38 were found eligible to participate in the intervention. Those who were ineligible were excluded most often due to lack of HDP diagnosis (n=13), being greater than 6 months postpartum (n=24), or self-reporting current CVD or taking antihypertensive medications (n=17). Of the 38 eligible, 23 consented to the intervention. Only 19 of the 23 consented to enroll in the study with 3 individuals no longer interested and 1 excluded due to activity (achieving >9000 steps/day). Two participants dropped out (n=1 moved out of the area and n=1 lack of time; attrition=11%) during the intervention, leaving 17 participants who successfully completed the intervention.

### Participants

Participants were on average 30.3 years of age (Table 1), and more often self-identified as White (14/17, 82%), were in a committed relationship (16/17, 94%), had earned at least a bachelor’s degree (9/17, 54%), and had an annual household income over US $100,000 (9/17, 54%). At the start of participation in the study, no individuals were on parental leave and were on an average of 4.1 (SD 0.81) months postpartum.

**Table 1.** Demographic characteristics of Hypertensive Disorders of Pregnancy Postpartum Exercise intervention participants^a^.

Participant characteristics (N=17)	Values
Age (years), mean (SD)	30.3 (3.6)
**Race, n (%)**	
White	14 (82)
Hispanic or Latina	1 (6)
Black	2 (12)
**Marital status, n (%)**	
Married or in a committed relationship	16 (94)
Single	1 (6)
**Education, n (%)**	
High school graduate or equivalent	4 (24)
Associate’s degree	3 (18)
Bachelor’s degree	4 (24)
Master’s or doctoral degree	5 (30)
**Annual household income (US $), n (%)**	
<$25,000	2 (12)
$25,000‐$99,999	6 (35)
$100,000‐$149,999	6 (35)
>$150,000	3 (18)
Earned wages outside of home, n (%)	14 (82)
**Parity, n (%)**	
1 child	6 (35)
2 children	7 (41)
3 or more children	4 (24)
**Hypertensive disorder of pregnancy, n (%)**	
Gestational hypertension	11 (65)
Preeclampsia	6 (35)
Months postpartum at start of intervention, mean (SD)	4.1 (0.81)

a Prior to the start of the intervention, participants completed an online survey to self- report their demographic characteristics. Continuous metrics were reported as mean (SD) and categorical variables as frequencies, n (%).

### Feasibility

Of all the participants who completed pretesting (n=19), 140 of the 152 (92%) sessions were completed. Of participants who completed the intervention (N=17), all 136 (100%) possible health coaching sessions were completed. Completers wore the Fitbit on 96% of all possible days. Almost all BP measures were obtained from completers with 90% of the pretest, 85% of week 5, and 85% of the posttest measures taken.

### Acceptability

Participants evaluated the intervention and components of the program including health coaching sessions, observed changes in their PA behaviors, changes in health and well-being, use of information sheets, Fitbit, activPAL, and BP monitors (Table 2). All completers (N=17) indicated they were satisfied with the program (100%) and would recommend this program to others (100%).

**Table 2.** Program evaluation of the Hypertensive Disorders of Pregnancy Postpartum Exercise intervention (N=17)^a^.

Item	Agree, n (%)	Neutral, n (%)	Disagree, n (%)
**Health coaching**			
The health coaching sessions helped me increase my physical activity	17 (100)	0 (0)	0 (0)
The health coaching sessions helped me decrease the amount of time I spend sitting	16 (94)	1 (6)	0 (0)
I felt supported by my health coach	17 (100)	0 (0)	0 (0)
My health coach was able to answer my questions	17 (100)	0 (0)	0 (0)
My health coach helped me understand how to perform physical activity safely	17 (100)	0 (0)	0 (0)
My health coach helped me create solutions to problems I faced	16 (94)	1 (6)	0 (0)
I felt like it was easy to talk to my health coach	17 (100)	0 (0)	0 (0)
I had enough time for my health coaching sessions	16 (94)	1 (6)	0 (0)
I wish I had more sessions with my health coach	8 (47)	8 (47)	1 (6)
I wish I had fewer sessions with my health coach	11 (65)	6 (35)	0 (0)
The length of time between sessions (weekly, every other week, and monthly) was appropriate	17 (100)	0 (0)	0 (0)
I enjoyed the ability to have health coaching sessions virtually from the comfort of my own home	17 (100)	0 (0)	0 (0)
**Movement behaviors**			
I feel like I have become more physically active	17 (100)	0 (0)	0 (0)
I know how to be physically active	17 (100)	0 (0)	0 (0)
I sit less during the day	17 (100)	0 (0)	0 (0)
I think that physical activity is an important part of living a healthy lifestyle	17 (100)	0 (0)	0 (0)
I am confident in my ability to stay physically active	17 (100)	0 (0)	0 (0)
**Health and well-being**			
I feel healthier	15 (88)	2 (12)	0 (0)
I have more energy during the day	13 (77)	2 (12)	2 (12)
I feel supported to live an active lifestyle	15 (88)	2 (12)	0(0)
I have noticed an improvement in my sleep	9 (53)	7 (41)	1 (6)
I have noticed an improvement in my mood	15 (88)	2 (12)	0 (0)
**Information sheets**			
I referenced the information sheets during the intervention	14 (82)	1 (6)	2 (12)
The information sheets helped me increase my physical activity	10 (59)	4 (24)	3 (18)
The information sheets were difficult to understand	3 (18)	7 (41)	7 (41)
**Fitbit wear**			
It was easy to set up my Fitbit (downloading app and linking device)	17 (100)	0 (0)	0 (0)
My Fitbit was easy to use	16 (94)	1 (6)	0 (0)
I checked the step count on my Fitbit daily	15 (88)	1 (6)	1 (5.9)
My Fitbit was uncomfortable to wear	4 (24)	2 (12)	11 (65)
The Fitbit app was easy to use	17 (100)	0(0)	0(0)
I checked my Fitbit app daily	9 (53)	5 (29)	3 (18)
The instructions given to me on how to use my Fitbit were helpful	15 (88)	1 (6)	0 (0)
It was difficult to remember to wear my Fitbit daily	8 (47)	9 (53)	0 (0)
It was difficult to remember to sync my Fitbit daily	2 (12)	4 (24)	11 (65)
I remembered to charge my Fitbit regularly	13 (77)	3 (18)	1 (6)
My Fitbit helped me achieve my step goals	15 (88)	2 (12)	0 (0)
**ActivPAL wear**			
The thigh activity monitor was uncomfortable to wear	3 (18)	5 (29)	9 (53)
It was easy to remember to complete my sleep log daily	8 (47)	5 (29)	4 (24)
I always completed my sleep log at the same time of day	6 (35)	4 (24)	7 (41)
**Blood pressure measurements**			
It was easy to remember to take my blood pressure	8 (47)	4 (24)	5 (29)
It was easy connecting my monitor to my phone	14 (82)	2 (13)	1 (6)
I know how to take a blood pressure correctly	17 (100)	0 (0)	0 (0)
It was useful knowing my blood pressure	16 (94)	0 (0)	1 (6)
It was hard to find time to take my blood pressure	2 (13)	7 (44)	7 (44)
**Overall program rating**			
I was satisfied with the program	17 (100)	0 (0)	0 (0)
I would recommend this program to others	17 (100)	0 (0)	0 (0)

a Participants ranked intervention features on a 5-point Likert scale from (1) “strongly agree” to (5) “strongly disagree.” Items were assessed at the end of the health coaching intervention via an online survey. To improve readability, (1) “strongly agree” and (2) “agree” were combined, as well as (4) “disagree” and (5) “strongly disagree”.

Concerning specific components of the intervention, all agreed that health coaching sessions helped increase their PA (n=17, 100%) and most agreed these sessions helped decrease their sitting time (n=16, 94%). Most participants said they referenced the information sheets during the program (n=14, 82%) and that this information helped them increase their PA (n=10, 59%). The Fitbit overall was rated as easy to use (n=16, 94%) and set up (n=17, 100%). Most said they checked their Fitbit step count daily (n=15, 88%). Most said Fitbit instructions were easy to understand (n=15, 88%), Fitbit was easy to remember to charge (n=13, 77%), and it helped them achieve their step goals (n=15, 88%).

All participants indicated they knew how to take a BP correctly after starting the program (n=17, 100%), with a majority indicating it was useful knowing their BP (n=16, 94%) and that it was easy to connect their monitor to their phone (n=14, 82%). Less than half of the participants agreed it was easy to remember to take their BP (n=8, 47%) and find time to do so (n=7, 44%).

### Preliminary Efficacy

Participants on average wore the activPAL for 7.39 (SD 0.51) days at pretesting and 6.55 (SD 1.46) days at posttesting. As seen in Table 3, activPAL measures did not significantly change from pre- to posttesting, including average steps achieved per week (*P*=.75), standing time (*P*=.78), stepping time (*P*=.49), and sitting time (*P*=.38). Fitbit data further contextualized the changes observed in steps during the intervention period. A statistically significant increase in Fitbit steps was only observed from pretesting to the end of the intensive intervention (*P*=.01).

**Table 3.** Physical activity and blood pressure changes of Hypertensive Disorders of Pregnancy Postpartum Exercise intervention participants (N=17)^a^.

Variable	Pretest	Week 5	Posttest	Hedges *g*
**ActivPAL measures**				
Steps, mean (SD)	6505.17 (2133.40)	—^b^	6643.60 (1997.68)	0.75
Standing time, mean (SD)	249.57 (65.40)	—	269.52 (113.51)	0.78
Stepping time, mean (SD)	88.57 (27.07)	—	91.93 (21.53)	0.49
Sitting time, mean (SD)	868.14 (189.03)	—	899.85 (255.90)	0.38
**Fitbit measures**				
Steps, mean (SD)	6584.47 (2705.65)	7898.27 (2359.32)^c^	7031.67 (2288.79)	0.48
**BP^d^ measures**				
Systolic BP, mean (SD)	122.12 (8.04)	120.67 (8.33)	120.83 (8.10)	–0.26
Diastolic BP, mean (SD)	82.95 (6.02)	81.77 (6.31)	81.15 (5.47)	–0.44
Hypertensive, n (%)	11 (64.7)	10 (58.8)	7 (41.2)^c^	–0.51

a All steps reported in units of average steps per week, time variables in average minutes per day, and blood pressures in average mm Hg. The activPAL4 micro and Fitbit Inspire 2 assessed changes in participants’ physical activity. The Omron Series 5 blood pressure monitor assessed changes in participants’ blood pressure. Reported Hedges *g* are effect size estimates between pretest and posttest. Given all variables were nonparametric in nature, Wilcoxon signed-rank test was used to detect significant differences between 2 timepoints. Statistical significance defined as *P*<.05.

b Not applicable.

c Significant difference from baseline.

d BP: blood pressure.

Changes in PA determinants, self-efficacy, planning, monitoring, and identity from pre- to posttesting are shown in Table 4. Overall, PA planning behaviors increased significantly (mean 5.35, SD 4.97 to mean 15.06, SD 3.09; *P*<.001), with increases in participant’s ability to plan when to be active, where to be active, who to be active with, how to overcome disruptions of plans and plan around difficult situations. Behaviors associated with monitoring of PA also significantly increased (mean 7.29, SD 3.44 to mean 13.00, SD 2.45; *P*<.001), with participants indicating increases in monitoring whether they are active enough, that they walk enough steps per day, and the awareness of PA goals. Components of PA identity significantly increased, including being more likely to identify as an active person, describing self to others as being active, needing PA to feel good, and indicating that giving up exercise would cause a sense of loss. No other statistically significant changes were observed.

**Table 4.** Change in physical activity determinants, self-efficacy, planning, monitoring, and identity of participants in the Hypertensive Disorders of Pregnancy Postpartum Exercise intervention (N=17)^a^.

Items	Pretest	Posttest	*P *value
**Planning physical activity, mean (SD)**			
I can plan when to be active	0.77 (0.75)	2.18 (0.39)	<.01
I can plan where to be active	0.82 (0.95)	2.12 (0.49)	.01
I can plan how to be active	0.65 (0.79)	2.29 (0.47)	.06
I can plan how often to be active	0.65 (0.70)	2.17 (0.64)	.06
I can plan who to be active with	0.65 (0.86)	1.65 (0.79)	.01
I know what to do if my plans are disrupted	0.65 (0.61)	1.41 (0.80)	.02
I can cope with setbacks	0.59 (0.51)	1.59 (0.62)	.10
I can plan around difficult situations	0.59 (0.51)	1.65 (0.79)	.04
Total planning	5.35 (4.97)	15.06 (3.09)	<.001
**Monitoring physical activity, mean (SD)**			
I monitor whether I am active enough	1.00 (0.71)	2.12 (0.60)	.01
I monitor that I walk enough steps per day	0.76 (0.56)	2.00 (0.87)	.02
I have physical activity intentions on my mind	1.82 (0.64)	2.41 (0.51)	.09
I am aware of my physical activity goals	1.12 (0.86)	2.12 (0.70)	.02
I try to be physically active regularly	1.35 (0.93)	2.18 (0.39)	.17
I try to achieve my physical activity goals	1.24 (0.90)	2.18 (0.39)	.21
Total score	7.29 (3.44)	13.00 (2.45)	<.001
**Physical activity identity, mean (SD)**			
I am an active person	1.88 (0.86)	2.18 (0.95)	.02
I describe myself to others as active	0.88 (0.70)	1.53 (1.23)	.04
I have goals related to physical activity	2.00 (1.28)	2.77 (0.66)	.94
Physical activity is central to my self-concept	1.53 (1.07)	1.82 (1.07)	.19
I need physical activity to feel good about myself	2.47 (1.01)	2.35 (1.06)	.04
Others see me as an active person	1.52 (1.01)	1.76 (1.15)	.47
Being active is more than just exercise	2.71 (1.21)	2.71 (0.92)	.32
I would feel loss if I had to give up exercising	1.94 (0.97)	2.29 (1.21)	.001
I think about physical activity often	2.29 (1.10)	2.29 (0.99)	.13
Total score	17.24 (6.88)	19.71 (6.55)	.09

a Participants completed online surveys prior to the start and at the conclusion of the intervention. Validated surveys assessed participants’ ability to plan for physical activity, monitor physical activity levels, and physical activity related identities. Differences assess in continuous measures with paired *t* tests and categorical with Fisher exact tests. All Likert scales ranged from “strongly disagree” (0) to “strongly agree” (5). Statistical significance defined as *P*<.05.

At pretesting, the average SBP was 122.12 (SD 8.04) mm Hg and DBP was 82.95 (SD 6.02) mm Hg (Table 3). Of the pretest readings, 11 (65%) participants had average readings that would be classified as HTN based on American Heart Association guidelines. Significant decreases in the number of participants with hypertensive readings were observed between pre- and posttesting (11 vs 7 participants; *P*=.01; Hedges *g*=–0.51). No statistically significant changes in SBP (mean –1.44, SD 4.68 mm Hg; *P*=.22) and DBP (mean –1.19, SD 4.76 mm Hg; *P*=.32) were observed from pretesting to the end of the intensive intervention (week 5) or from pretesting to posttesting (SBP: mean –1.28, SD 3.59; Hedges *g*=–0.26; *P*=.16 and DBP: mean –1.80, SD 5.03; Hedges *g*=–0.44; *P*=.12). When assessing the association between an increase in steps by 1000 and BP, no findings were statistically significant (SBP: mean –0.30, SD 1.11 mm Hg; *P*=.79 and DBP: mean –1.52, SD 1.46 mm Hg; *P*=.32).

## Discussion

### Principal Findings

The HyPE intervention included 17 participants whose prior pregnancies were complicated by HDP. Overall, the intervention was found to be feasible and acceptable as we hypothesized. Preliminary efficacy assessments did not support our hypothesis as participants did not significantly increase the number of activPAL measured steps achieved from pre- to posttesting. Indicating that we failed to achieve a clinically significant impact on the behavioral target, a critical milestone in phase IIa of the ORBIT model [16]. Factors promoting PA (eg, PA monitoring, planning, and identity) were observed to significantly improve. HTN prevalence was the only BP metric observed to significantly decrease from pre- to posttesting.

### Contextualizing Findings

CVD has previously been described as a “man’s disease,” but the acknowledged importance of CVD in women is increasing. Reviews of CVD-related clinical trials have highlighted an underrepresentation of women in research [34], subsequently attributing to sex-based disparities in the clinical guidelines, recommendations, treatments, and health outcomes related to CVD. Potential downstream effects of these disparities were reflected in our findings, with 94% (n=16) of individuals reporting at no time did a health care provider discuss the increased risk of CVD associated with HDP diagnosis. With HDP an important risk factor for CVD, it is critical as a field to find effective strategies to promote cardiovascular health in this high-risk population.

To the authors’ knowledge, only 1 randomized clinical trial has assessed the efficacy of a PA intervention after HDP. Participants of that study increased their steps more than the control (mean difference: 647 steps per day, 95% CI 169‐1124; *P*=.009) [35]. A total of 65% of participants completed the study with 94% indicating that they would recommend the program to others [35]. The HyPE intervention had similar adherence and acceptability results, with 90% of participants completing the intervention and 100% satisfied with the program. Intervening on PA this early in postpartum (3‐6 months postpartum) is novel and our results indicate that interventions during this timeframe may be feasible and acceptable. Although we did not observe significant increases in device-measured PA, participants did significantly increase their self-reported abilities to plan and monitor PA. This discrepancy may be a result of seasonality, given that the winter season is associated with a decrease of up to 2500 steps compared to summer months [36]. Those completing their posttesting in the winter (ie, December or January; n=9, 53%), consequently may have seen a decrease in their steps due to the colder temperatures. While participants in the HyPE intervention did not increase their steps, the intervention may have prevented the typical decline observed when transitioning from summer to winter months. Future interventions should consider the impacts seasonality may have on their population and develop strategies to combat potential effects colder weather may bring. Finally, we included the target population in the development of the intervention in previous formative work assessing the unique behavioral determinants, needs, and desires of postpartum individuals with HDP [17]. Formative work has been called for in postpartum PA intervention development with inclusion of the target population in this process essential to creating equitable and culturally appropriate solutions [37,38].

### Limitations

There are several limitations of this study to consider when interpreting results. There was no control group due to this being a proof-of-concept study, limiting the ability to assess whether the changes observed were a direct effect of the intervention itself. This decision was intentional, however, as very few PA interventions have targeted the early postpartum period, and demonstrating feasibility and acceptability was our primary objective. This also follows the ORBIT model, representing phase IIa in the behavioral treatment developmental pathway [16]. Given that the milestone for successful completion of phase IIa was not reached with no clinically significant changes in behavior detected, further refinement and optimization of the intervention is warranted. These refinements should include lowering the <9000 step inclusion criteria threshold, as well as the duration or frequency of study visits. In addition, there was no activPAL4 micro assessment of PA at the end of the intensive intervention (week 5), which limited our ability to determine the immediate effects of the intervention on PA behavior. It is also possible that our PA eligibility criteria (must achieve <9000 steps/day) may have biased our results by not recruiting a truly inactive population. It is estimated that achieving Americans 7000‐8000 steps is equivalent to meeting the PA guidelines for Americans [39]. Further, the significant decrease in hypertensive individuals should be interpreted with caution, as this may reflect that most individuals who were classified as hypertensive were only slightly above the American Heart Association defined BP threshold for HTN. Finally, the population recruited was not racially, ethnically, or socioeconomically diverse, with a majority of individuals being White and of high socioeconomic status. Future studies will require a larger, more diverse sample and should consider recruiting individuals achieving <7000 steps per day. Future studies are needed to contextualize the needs and desires of diverse populations. Given the importance intersectional identities may have in a birthing person’s health and experiences with the medical system, the inclusion of diverse cohorts is essential to the equitable promotion of female cardiovascular health after HDP.

### Conclusions

HDP incidence is increasing and is associated with an increased risk of CVD later in life. Our study suggests that intervening in PA behaviors early after HDP is feasible and acceptable. Future studies should further refine intervention strategies for this population and test its efficacy in a larger, more diverse, and less active sample.

## Abbreviations

BP: blood pressure
CVD: cardiovascular disease
DBP: diastolic blood pressure
HDP: hypertensive disorders of pregnancy
HTN: hypertension
HyPE: Hypertensive Disorders of Pregnancy Postpartum Exercise
ORBIT: Obesity-Related Behavioral Intervention Trials
PA: physical activity
SBP: systolic blood pressure
